# Letter on “Sharing trial results directly with trial participants and other stakeholders after the SARS-CoV-2 pandemic hit the UK – experience from the ActWELL trial”

**DOI:** 10.1186/s13063-021-05340-3

**Published:** 2021-06-05

**Authors:** Hanne Bruhn, Annie S. Anderson, Amy Hickman, E. Jane Macaskill, Shaun Treweek

**Affiliations:** 1 Health Services Research Unit, University of Aberdeen, Room 306, 3rd Floor, Health Sciences Building, Foresterhill, Aberdeen, AB25 2ZD UK; 2 Centre for Research into Cancer Prevention and Screening, University of Dundee, Ninewells Hospital & Medical School, Dundee, DD1 9SY UK; 3 Breast Cancer Now, 222 Leith Walk, Edinburgh, EH6 5EQ UK; 4 Department of Breast Surgery, Level 6, Ninewells Hospital and Medical School, Dundee, DD1 9SY UK

**Keywords:** Randomised trials, Research transparency, Result dissemination, Trial conduct, Online events, COVID-19

## Abstract

After the SARS-CoV-2 pandemic took hold in the UK, the ActWELL trial team’s plans to present the trial results to participants and other stakeholders had to change. Instead of face-face events, three online events were planned and hosted successfully. In this article, we describe the choices made in planning and organisation of the online events including things we would do differently if we were to do it again. We think that online events are a useful platform when informing participants and other stakeholders of the results of your trial, even beyond the SARS-CoV-2 pandemic, and we hope this article can help other trial teams to plan their own online events.

**Supplementary Information:**

The online version contains supplementary material available at 10.1186/s13063-021-05340-3.

## Background

In 2015, the United Kingdom’s (UK) Health Research Authority (HRA), whose core purpose is “to protect and promote the interests of patients and public in health and social care research”, published guidance [1] recommending that all researchers offer trial results to trial participants. Following this guidance, the ActWELL (ISRCTN11057518) [2] trial team planned to share the trial’s results with participants. The ActWELL trial results were published in March 2021 [3].

The ActWELL multi-centre RCT evaluated the effect of a theory-based lifestyle intervention (delivered via two, face-to-face visits and up to 9 telephone calls with a Breast Cancer Now (BCN) lifestyle coach) on changes in weight and physical activity over a 1-year period. Women were offered the chance to participate when they attended their routine National Health Service (NHS) Breast Screening appointment in four Scottish health boards. The intervention itself was delivered by BCN volunteer lifestyle coaches who were recruited and managed by the charity.

As the trial drew to a close, a researcher was appointed in July 2019 to work on a dissemination package, which included preparing written summaries of results and organising face-to-face dissemination events in each of the four Scottish health boards where recruitment had taken place. The SARS-CoV-2 pandemic changed these plans.

Here we present the practical considerations we faced when switching from face-to-face meetings to three online events for feeding back trial results to participants and other stakeholders during the SARS-CoV-2 pandemic. We also consider aspects we would likely do differently next time based on attendee feedback.

We hope the article will provide a practical guide to conducting online dissemination events for trial participants, an activity that will remain important beyond the SARS-CoV-2 pandemic.

## Approach

### Purpose of the events

The purpose of the online events was twofold:

1. To inform trial participants and other stakeholders of the trial findings
2. To enable attendees to ask questions about any aspect of the trial, its results and implications arising from the findings

### Event programme

The programme for the events was the same for all three events and each event lasted for about 90 min. Four trial team members spoke for about 60 min in total, followed by a 5-min break and 25–30 min for a facilitated question and answer session (see supplementary file 1).

### Running of events

We decided to plan three events with the intention of having more if the dates we proposed left many participants still unable to attend. The events were arranged for different days and different times—one lunchtime (12.30–14.00) and two evenings (18.30–20.00). Events were delivered in English.

Each event was limited to 100 attendees in order to allow the sessions to be interactive and manageable from the trial team’s perspective. Registration and waiting lists were managed through Eventbrite (https://www.eventbrite.co.uk/), which is free to use for free events such as ours. The Eventbrite event page could be kept private so only people with a link were able to register.

### Stakeholders/attendees

All women taking part in the ActWELL trial and who had not withdrawn or moved away from Scotland were offered an invitation to the online events. ActWELL participants were women eligible for the UK breast screening programme (i.e. aged 50–70 years), with a mean age at baseline of 59.1 years (SD 5.44). Trial participants came from all socio-economic groups, with 16% from SIMD 1 and 2 (areas of highest social deprivation) in Scotland. Most women were of White British ethnicity (95%).

Other stakeholders including BCN lifestyle coaches, breast screening centre staff and leisure centre staff were also identified by the trial team and invited by email. Email addresses were available for most trial participants; those without an email address were sent postal invitations.

### Invitations and permissions

One invitation was designed for all stakeholders and for both email and postal distribution (see supplementary file 2). Due to data protection laws, the BCN volunteer lifestyle coaches had to be invited by BCN staff who held their contact details. Dissemination of trial results to participants, and holding dissemination events had been included in the initial NHS REC review. Our events were held after the formal end of the ActWELL study and we confirmed with the University of Dundee sponsor representative that the proposed events were acceptable. Anyone interested in attending was asked to register for the event on Eventbrite.

### Platform and event management

Two hosting platforms were considered for the events: Microsoft Teams (Meetings/Live; https://www.microsoft.com/en-gb/microsoft-teams/group-chat-software) and Zoom (Large meeting/Webinar; https://www.zoom.us). The platform had to enable an interactive session and we chose the Zoom Large Meeting add-on. Many members of the public are now familiar with Zoom, there is evidence that research participants prefer it to other platforms [4], and with the large meeting add-on, it was possible to let attendees ask questions both via chat and audio. Further benefits of using Zoom were that it integrates well with Eventbrite and enables you to communicate with your registered attendees, as well as send out an Evaluation survey using Survey Monkey (https://www.surveymonkey.co.uk). There was some initial resistance from our institution (the University of Aberdeen) to give approval for Zoom because its institutional platform is Teams and because of data security fears. However, concerns about Zoom security fears in October 2020 were reduced compared to earlier in 2020, especially with Zoom’s use of passwords and Waiting room. Evidence that research participants preferred Zoom was also persuasive. Permission to use Zoom for these events was therefore granted by the University.

Event management and technical assistance was hired for the events so that the team had someone who was very familiar with the platform and who was able to “produce” the event and help it run smoothly as well as to record it (https://stauntonmedia.ie/). The sessions were recorded mainly for internal learning purposes and with a view to possibly make it available to participants who could not attend events if requested. Otter.ai (https://otter.ai) was used for live captioning of the events as it integrates with Zoom to provide captions in a separate browser window.

Event management also provided help for attendees with joining and other technical issues, which meant they could contact a member of the team behind the scenes via email.

### Evaluation

To get feedback from attendees and to help us make suggestions for improvements for future events, we sent attendees an evaluation survey after the event (see supplementary file 3). The survey included seven questions with a mixture of closed and open-ended text responses.

## Implementation

The ActWELL trial was led by Professor Annie S. Anderson (ASA) from the University of Dundee, and contact details for trial participants were held by the Health Informatics Centre (HIC) at the University. Invitation distribution was therefore managed through HIC, which charged the trial team to do this as it was not part of the original contract between the trial team and HIC. Other stakeholders were sent an invitation via email by members of the trial team (Table 1).

**Table 1:** Stakeholder groups and the numbers invited, registered to attend, attending the events and responding to the event evaluation survey

Stakeholder group	Invited	Registered (%)	Attended (% of those registered)	Responded to evaluation survey (% of those attending)
Trial participants^a^	438	79 (18)	53 (67)	30 (56)
Breast Cancer Now (BCN) coaches	45	22 (49)	21 (95)	12 (57)
NHS staff^b^	12	8 (67)	8 (100)	3 (38)
Leisure Centre staff	8	2 (25)	2 (100)	1 (50)
BCN staff	5	5 (100)	4 (80)	0
Public representatives	5	1 (20)	1 (100)	1 (100)
Funder staff	2	0	0	0
Trial team	3	0	0	0
Total	519	117 (23)	89	47

^a^All participants within ActWell who are alive, have not withdrawn, have not left Scotland and have the status “Completed Study”

^b^Twelve NHS staff members were invited directly (investigators and their team) while staff at breast cancer screening centres were sent invitations via a generic email address and the number of recipients is therefore unknown

The invitations were emailed (n = 400) and posted (n = 38) out 15 days before the first event. We were fortunate that most participants (91%) had provided their email address and by proxy could be expected to have internet access. One trial participant emailed to say she did not want to attend the online event but would appreciate a written summary of the results. Out of 519 stakeholders invited, a total of 89 attended of which 53 were trial participants and 21 BCN lifestyle coaches. The lunchtime event was the most popular with the highest number of registrations (n = 50), including most NHS Staff attending and a total of 39 attendees.

A number of attendees were in contact via email before and after events. One of the trial participant attendees sent a list of questions providing us with a useful set of questions to start off each Q&A session. Other email contacts were mainly about how to access the results other than at the events, issues around joining the events and thanking the trial team for holding the events. Demand was met with the three events planned and therefore no further events were scheduled.

### Experience of the event

All three events were delivered as planned and there were no technical problems. All three were delivered as per the programme shown in Supplementary 1. All three started and finished on time.

The majority of survey respondents indicated that they had had all their questions about the ActWELL study and its findings answered. Five of the 47 attendees who responded said that their questions had not been answered and these questions were around future plans for the rollout of the intervention, which the team could not yet answer (n = 2), making the intervention more accessible to all socio-economic groups (n = 1) and specific risk (n = 2). In addition, one attendee also requested access to the paper when published in their response.

All survey respondents indicated that the information was presented in a way that was easy to understand (n = 47). One did add a comment that there was not enough time to look at the results on the slides.

All responses to the survey are summarised in Supplementary file 4.

From our perspective as speakers (ASA, AH, JM and ST), the online events were a great success and hugely rewarding. Being able to speak directly to participants and others who had helped us to do the trial was a joy and the obvious interest and appreciation of those attending made it all the more satisfying. We were asked many excellent and probing questions in over 90 min of questioning across the three events and these both made us think and gave us confidence that the ActWELL intervention [5] is worth pursuing. It is hard to now imagine not including online dissemination events in future trial dissemination plans.

### Costs

The total cost for the three events was £1,624 GBP (€1,842 EUR; $2,220 USD) with trial participant invitations and event management and technical assistance at the events being the highest expenditure items (see supplementary file 5). Staff hours spent planning and organising the events have been counted separately (see supplementary file 5) as the cost depends on local circumstances. A total of 40 h, over 2 months, were spent by the research team on preparing the events. Having now done these events, we would expect somewhat less time to be needed for future events.

## Recommendations

There is clearly an appetite among trial participants and other stakeholders for events of this type and attendance after registration was better than is usual (in personal experience) for other academic online events. Overall, all the events were well received and there are only a few things we might do differently next time.

Although the evaluation was overwhelmingly positive, we think it would have been useful to include Patient and Public Involvement (PPI) partners in all of the planning process for online events in line with current best practice recommendations from the HRA [6]. Unfortunately, none of the PPI partners who had been involved in ActWELL was available for ours and it did not seem fair to expect someone new to the project to get involved this late in the trial and under considerable time pressure. For future events, it will be particularly important to consult PPI partners about the best way to deliver trial results to participants. Three participants expressed a wish the events had been face-to-face in the evaluation although we do not know whether lockdown will have made the online event more attractive in the future. Our key learning point is that trial teams should plan from the start to offer both online and face-to-face delivery of trial results to participants unless PPI partners say otherwise.

For future events, we would consider changing the programme to give a little more focus on the results themselves and less on the background to the trial. We would also consider offering a slightly later event start for at least one event, so 19.00 rather than 18.30 start. Considering the number of professional stakeholders who attended the events, also in their own time, we would in future consider offering a “quick” lunchtime session specifically aimed at them. Alternatively, by providing more timing detail in the joining instructions, attendees could choose to join the event at the point they were most interested in (e.g. when the results were presented) if they were less interested in other parts of the session.

We would recommend the use of an event management and technical assistance company, or your own organisation’s event management staff if available. Having professional support completely removed any concern about the smooth running of the event and allowed speakers to concentrate entirely on presenting and discussing the trial with attendees. The cost of £770 was modest when compared to the cost of organising three face-to-face events (e.g. the original main face-to-face event was costed at £8000 (€9252 EUR; $11,154 USD) in total including travel for study team and attendees, overnight accommodation for the study team, venue and catering during the event).

After several queries from participants, we will now offer the results in three different modes, not just the two that were planned—dissemination events, written plain English summary and now also a recording of dissemination events. All three will be available on a trial-specific website, which we will tell participants about once it becomes live (www.actwellstudy.org). Participants will be emailed or mailed the link to the website, so it is their choice whether to access the information or not. Other people, including NHS staff, emailed to ask if the event was recorded and whether they could see it as they missed or were not available for the live events. This will also be of benefit to any attendees with internet connection issues and those who want to go over the results again at their own pace.

## Summary

Providing trial results direct to trial stakeholders online was a success and should also be considered post-pandemic. Face-to-face events will still be important but we suggest that online events should always be offered because they remove the need to travel (which may be a problem for some trial participants), are easily recorded and are efficient in terms of time and cost. However, trial teams will have to consider how to communicate with and disseminate to stakeholders who do not have internet access, or who have access but are less familiar with internet-based tools.

Lastly, there is a considerable workload associated with planning dissemination events, whether face-to-face or online, which should not be underestimated. We hope this article will give pointers as to what trial teams need to plan and budget for when designing their trials. This work is worth it.

We will give the last word to a participant from the ActWELL comparison group who attended the online event of 26 November 2020:

It was tremendous to have the Feedback Day, far beyond what I would have expected (so much more than an A4 sheet mailed out or even less). It was also a valuable reminder to me as to why I should maintain the changes and some of the techniques for achieving them. (S. MacAskill, with permission).

## Supplementary Information

**Additional file 1.** Plan and programme for ActWELL online dissemination meetings Nov 2020.

**Additional file 2.** Email invitation to all potential attendees (stakeholders).

**Additional file 3.** Evaluation survey for ActWELL online dissemination.

**Additional file 4.** Summary of evaluation survey for ActWELL online events.

**Additional file 5.** Expenditure for ActWELL online events and Staff hours (1 person).

## Data Availability

Data sharing is not applicable to this article as no datasets were generated or analysed during the current study.

## Abbreviations

ActWELL: Working together to support *ACT*ive living and *WELL* being (ActWell) in the health promoting health service—feasibility trial to reduce breast cancer risk factors
BCN: Breast Cancer Now
HRA: Health Research Authority
RCT: Randomised controlled trial
