# Supplementary material for: Letter on “Sharing trial results directly with trial participants and other stakeholders after the SARS-CoV-2 pandemic hit the UK – experience from the ActWELL trial”
Source: Trials. 2021 Jun 5;22:381. doi: 10.1186/s13063-021-05340-3 (PMC8179700; doi:10.1186/s13063-021-05340-3)
Supplement: Supplementary file 1 — Additional file 1. Plan and programme for ActWELL online dissemination meetings Nov 2020. [file 13063_2021_5340_MOESM1_ESM.docx]

**Plan and programme for ActWELL online dissemination meetings Nov 2020**

Attendees join meetings 15 mins before start – ActWELL logo in waiting room

**Outline**

**18.30 Welcome and housekeeping (Annie)**

- Mic options, screen options, how & when, where to ask questions, session will be recorded but Q&A not, captions added.
- Outline to session.

**18.45 Background to ActWELL (Jane)**

- Why is ActWELL important from a clinical perspective?

18.55 – The intervention (Annie)

- The ActWELL intervention (and who did what) and why weight loss is important.

**19.00 Volunteer support for lifestyle change (Amy)**

- Learning about volunteer support for lifestyle change
- Volunteer delivery of the intervention

**19.10 Dance Break – 5 mins**

**19.15 The results of the trial (Annie)**

- How much weight loss and physical activity change was there?
- Where there other changes?
- What participants, coaches, leisure centre and screening centre staff said about ActWELL
- Implications of the study findings for breast cancer risk reduction
- Implication of the study findings for breast screening

**19.25/19.30 Questions and discussion (Shaun to chair)**

**20.00 Thanks and close (Annie)**
