# Supplementary material for: Letter on “Sharing trial results directly with trial participants and other stakeholders after the SARS-CoV-2 pandemic hit the UK – experience from the ActWELL trial”
Source: Trials. 2021 Jun 5;22:381. doi: 10.1186/s13063-021-05340-3 (PMC8179700; doi:10.1186/s13063-021-05340-3)
Supplement: Supplementary file 2 — Additional file 2. Email invitation to all potential attendees (stakeholders). [file 13063_2021_5340_MOESM2_ESM.docx]

**Email invitation to all potential attendees (stakeholders)**

[Email subject line: Invitation to event about the ActWELL study findings]

[email/letter content:]

Dear [NAME],

The results for the ActWELL study are in! I’m pleased to invite you to join us for a free online event where the study findings will be presented by members of the study team, and you can ask any questions you might have about the study.

**Who is invited:** Anyone involved in the study – participants, BCN Volunteer Coaches, NHS Screening Centre staff, representatives from the funder, leisure centre staff.

**Speakers:** Four study team members will be talking about the ActWELL study

Annie Anderson, Professor of Public Health Nutrition and the Chief Investigator of the ActWELL study, University of Dundee

Jane Macaskill, Consultant Oncoplastic Breast Surgeon, NHS Tayside

Amy Hickman, Senior Public Health and Wellbeing Officer, Breast Cancer Now

Shaun Treweek, Professor of Health Services Research, University of Aberdeen

**When:** You have a choice of days and times

Tuesday 17^th^ November starting at 6.30 pm – 8.00 pm, get a ticket here: <https://www.eventbrite.co.uk/e/the-actwell-study-results-meeting-tickets-124702359113>

Thursday 19^th^ November at starting 12.30 pm – 2.00 pm, get a ticket here: <https://www.eventbrite.co.uk/e/the-actwell-study-results-meeting-tickets-124722661839>

Thursday 26^th^ November starting at 6.30 pm – 8.00 pm, get a ticket here:

<https://www.eventbrite.co.uk/e/the-actwell-study-results-meeting-tickets-124729231489>

**Registration:** You can register now and until the day before your chosen event but note that tickets are limited to 100 for each event.

Please only register for one event as all events will have the same programme.

**Where:** Please register for the event you want to attend and a link to Zoom will be made available on the day.

If you have not used Zoom before it is recommended to connect early as joining the first time takes a little while.

The event programme is also available on the registration page.

**Contact:** If you have any questions please contact Hanne Bruhn (hanne.bruhn@abdn.ac.uk).

I hope you will join me and the team in November!

Professor Annie Anderson

Chief Investigator of the ActWELL study
