# Supplementary material for: Letter on “Sharing trial results directly with trial participants and other stakeholders after the SARS-CoV-2 pandemic hit the UK – experience from the ActWELL trial”
Source: Trials. 2021 Jun 5;22:381. doi: 10.1186/s13063-021-05340-3 (PMC8179700; doi:10.1186/s13063-021-05340-3)
Supplement: Supplementary file 3 — Additional file 3. Evaluation survey for ActWELL online dissemination. [file 13063_2021_5340_MOESM3_ESM.docx]

**Evaluation survey for ActWELL online dissemination**

Survey questions:

1. Were all your questions about the ActWELL study and its findings answered? [Yes/No]

If no, which questions would you have liked to ask or which questions were not answered? [Open-ended]

1. Was the information presented in a way that was easy to understand? [Yes/No]

If no, what information or which part of the programme was hard to understand? [Open-ended]

1. How likely is it that you would recommend an event like this to a friend or colleague? [0-10 scale]
2. Overall, how would you rate the event? [Excellent, Very good, Good, Fair, Poor]
3. What did you like or dislike about the event? [Open-ended]
4. Was the event length too long, too short or about right? [Much too long, too long, about right, too short, much to short]
5. Is there anything else you’d like to share about the event? [Open-ended]
