# Supplementary material for: Letter on “Sharing trial results directly with trial participants and other stakeholders after the SARS-CoV-2 pandemic hit the UK – experience from the ActWELL trial”
Source: Trials. 2021 Jun 5;22:381. doi: 10.1186/s13063-021-05340-3 (PMC8179700; doi:10.1186/s13063-021-05340-3)
Supplement: Supplementary file 4 — Additional file 4. Summary of evaluation survey for ActWELL online events. [file 13063_2021_5340_MOESM4_ESM.docx]

**Summary of evaluation survey for ActWELL online events**

The majority of survey respondents indicated that they had had all their questions about the ActWELL study and its findings answered. Five of the 47 attendees who responded said that their questions had not been answered and these questions were around future plans for the rollout of the intervention which the team could not yet answer (n=2); making the intervention more accessible to all socio-economic groups (n=1); specific risk (n=2). In addition, one attendee also requested access to the paper when published in their response.

All survey respondents indicated that the information was presented in a way that was easy to understand (n=47). One did add a comment that there was not enough time to look at the results on the slides.

Overall, the majority rated the events as very good or excellent (n=42) while five rated the events as fair or good. Most thought the length of the events was ‘about right’ (n=39) while few thought the events had been too long (n=8).

In terms of what attendees liked or disliked about the events there were comments from 26 trial participants and 11 BCN coaches, three NHS staff and one leisure centre staff member. Trial participants were pleased to get the results and liked the interactive format of the events. In terms of more critical feedback

- A few would have liked to spend more time on the results themselves and not so much on the background to the trial.
- Some would have preferred a face-to-face event.
- One commented that the timing for the event was early (6.30pm) as they had just returned from work.
- A few requested access to written results, which was also promised by the team at the beginning on each event.
- One BCN lifestyle volunteer coach would have liked a more in-depth discussion of some of the issues and the time allocated was too short.
