# Supplementary material for: Letter on “Sharing trial results directly with trial participants and other stakeholders after the SARS-CoV-2 pandemic hit the UK – experience from the ActWELL trial”
Source: Trials. 2021 Jun 5;22:381. doi: 10.1186/s13063-021-05340-3 (PMC8179700; doi:10.1186/s13063-021-05340-3)
Supplement: Supplementary file 5 — Additional file 5. Expenditure for ActWELL online events and Staff hours (1 person). [file 13063_2021_5340_MOESM5_ESM.pdf]

Expenditure for ActWELL online events

Item	Cost
Postal invitations for ActWELL participants (n=38)<br>-printing and posting by HIC Dundee	£29.64
Email invitation to most participants (n=400 out of 464)<br>-combined cost for setting up and management of postal/email contact by HIC Dundee	£720.00
BCN email invitations to coaches (BCN/ActWELL Team)	£0.00
Email invitations to leisure centres (ActWELL Team)	£0.00
Email invitations of breast screening centres and mobile units (ActWELL Team)	£0.00
Registration management - Eventbrite (ActWELL Team)	£0.00
Event evaluation through Eventbrite and SurveyMonkey (ActWELL Team)	£0.00
Tech support at live events - Cormac Staunton (https://stauntonmedia.ie/)<br>-test, recording best practice; at event tech support, break music	£770.00
Live captioning using Otter.ai (https://otter.ai)<br>-Free one month trial but reduction for Higher Education Institutions also available	£0.00
Zoom pro licence for 2 months<br>-need organisation VAT number to pay	£23.98
Zoom large meeting add-on for 2 months<br>-need organisation VAT number to pay	£80.00
**TOTAL COST**	**£1,623.62**
**In addition staff hours should be added to the cost<br>See breakdown of staff hours on next page**	**40 hours**

Staff hours (1 person)

Activity	Comment	Estimated hours
Meetings with ActWELL lead during planning stage	Also via email	4
Quotes for tech help x3	3x meetings (various emails)	4
Platform - meeting about MS Teams	1xmeeting	1
Platform - institutional issues around Zoom	1xmeeting and emails	1
Drafting invitations and amendment after team review		1
Drafting evaluation plan and survey and amendments after team review		3
Drafting programme and discussion with team	meetings and email	1
Setting up Enventbrite pages x3 including Monkeysurvey	1xmeeting to test page and link	3
Prepare event PowerPoint Slides		3
Setup Zoom with Otter.ai		1
Liasing with HIC to send invitations to trial participants and arrange payment		1
Prepare mail merge invitations		1
Respond to emails	After invitaitons, during and after events	1
Arrange and attend test event for team		1
Attending events	30 mins. before, 30 mins. after event and survey distribution	9
Write report of events and evaluation survey	Clean evaluation data and import into SPSS, write syntax	5
**TOTAL HOURS**		**40**
